# Armored IL-18 CAR-T cells: bioinformatics-guided design for cold tumor infiltration

**DOI:** 10.1007/s10238-026-02284-3

**Published:** 2026-08-10

**Authors:** Muhammad Shahid Mehmood, Hikmat Ullah, Taimoor Wajid, Şehriban Büyükkılıç, Tabish Arif, Mohammad Azhar Kamal, Faizan Ali, Mohammad Jalal Nazari

**Affiliations:** 1 University of Agriculture Faisalabad, Faisalabad, 38000 Pakistan; 2 University of Chinese Academy of Sciences, Beijing, 100049 China; 3 King Edward Medical University, Lahore, Pakistan; 4 Dokuz Eylül University, 35340 Izmir, Turkey; 5 University of Management and Technology, Lahore, Pakistan; 6 Department of Pharmaceutics, College of Pharmacy, Prince Sattam Bin Abdulaziz University, Al-Kharj, 11942 Saudi Arabia; 7 Cholistan University of Veterinary and Animal Sciences, Bahawalpur, Pakistan; 8 Medical Research and Technology Center, Khatam Al-Nabieen University, Kabul, Afghanistan

**Keywords:** IL-18 armored CAR-T cells, Cold tumor microenvironment, Bioinformatics-guided CAR design, Solid tumor immunotherapy, Multi-omics cancer immunology

## Abstract

Chimeric antigen receptor (CAR) T cell therapy has shown remarkable efficacy in hematologic malignancies, yet its translation to solid tumors remains a fundamental challenge. Immunologically “cold” tumors, characterized by poor T cell infiltration, dense stromal barriers, and a profoundly immunosuppressive tumor microenvironment (TME), continue to resist conventional CAR-T cell approaches. Armored CAR-T cells engineered to secrete interleukin-18 (IL-18) represent a promising fourth-generation strategy designed to overcome these limitations. IL-18 operates at the interface of innate and adaptive immunity, driving M1 macrophage polarization, dendritic cell maturation, NK cell activation, and the induction of CXCL9/CXCL10 chemokine gradients that restore lymphocyte trafficking into tumor tissue. To guide the rational design of these constructs, bioinformatics pipelines play an increasingly central role. Multi-omics integration of transcriptomic, proteomic, and single-cell RNA sequencing data enables precise tumor antigen selection, epitope prediction, and structural optimization of CAR constructs using tools such as AlphaFold, RosettaDock, and NetMHCpan. In silico safety screening further evaluates off-target toxicity risks and cross-reactivity against healthy tissues. Controlled IL-18 delivery strategies, including NFAT-inducible promoters, SynNotch logic-gated systems, and Granzyme B-cleavable pro-IL-18 constructs, provide spatial and temporal safeguards against systemic hyperinflammation. Preclinical evidence from xenograft and syngeneic models demonstrates enhanced tumor control, sustained T cell persistence, and induction of endogenous anti-tumor immunity. This review synthesizes current evidence and highlights how bioinformatics-guided engineering of IL-18 armored CAR-T cells offers a translatable framework for converting cold tumors into immunologically responsive, therapeutically tractable targets.

## Introduction

Chimeric antigen receptor (CAR) T cell therapy has fundamentally transformed the treatment of hematologic malignancies, but its application in solid tumors remains limited [1]. Over the past three decades, CAR constructs have undergone progressive architectural evolution. First-generation CARs relied exclusively on the CD3 intracellular signaling domain, resulting in insufficient in vivo persistence [2]. Second- and third-generation CARs focused on this limitation by incorporating one or two costimulatory domains, such as CD28, 4-1BB, or OX40, thereby recapitulating the physiological “Signal 2” required for full T cell activation [3]. Notably, the choice of costimulatory domain dictates distinct metabolic programs: CD28 signaling promotes PI3K/Akt-driven aerobic glycolysis and rapid effector differentiation, whereas 4-1BB favors fatty acid oxidation and oxidative phosphorylation (OXPHOS) [2], supporting mitochondrial biogenesis and long-term persistence [4]. More recently, fourth-generation TRUCKs (T cells Redirected for Universal Cytokine-mediated Killing) and fifth-generation CARs integrating truncated cytokine receptor domains to activate JAK–STAT signaling have been developed to overcome the hostile solid tumor microenvironment [5, 6].

The limited efficacy of CAR-T cells in solid tumors arises from a combination of physical and immunological barriers. Dense extracellular matrix (ECM) structures, cancer-associated fibroblast (CAF)-mediated exclusion, and abnormal vasculature collectively impair T cell trafficking and infiltration [7]. Within the tumor microenvironment (TME), CAR-T cells encounter potent immunosuppressive networks composed of regulatory T cells, myeloid-derived suppressor cells, and inhibitory cytokines such as TGF-β and IL-10, as well as adenosine signaling via the CD39/CD73 axis [1, 5]. Chronic antigen exposure in this context promotes T cell exhaustion, characterized by stable epigenetic reprogramming and upregulation of inhibitory receptors, including PD-1, LAG-3, and TIM-3 [8]. In parallel, intratumoral heterogeneity drives antigen escape, allowing antigen-negative tumor clones to expand under selective immune pressure [9].

Solid tumors can be immunologically classified as inflamed, immune-excluded, or immune desert phenotypes. The letter defines “cold” tumors, in which T cells are either absent or confined to stromal regions without effective tumor penetration [10]. These tumors typically exhibit low neoantigen burden and impaired antigen presentation, often through β2-microglobulin loss and downregulation of MHC molecules. Additionally, defective chemokine signaling, such as insufficient CXCL9 and CXCL10 gradients-limits T cell recruitment, while stromal sequestration further restricts immune cell access to malignant cells [11, 12].

To overcome these barriers, armored CAR-T cells were developed based on the TRUCK concept, enabling antigen-dependent delivery of transgenic cytokines as an engineered [13]. Different cytokines confer distinct functional advantages: IL-12 induces strong innate immune activation but is limited by systemic toxicity; IL-15 enhances CAR-T cell persistence and promotes a stem-like memory phenotype; and IL-18 provides balanced immunomodulatory activity [14]. By activating macrophages, natural killer cells, and CD8⁺ T cells, IL-18 functions at the interface of innate and adaptive immunity [15]. This cytokine armoring strategy facilitates remodeling of the immunosuppressive TME and reduces antigen escape by recruiting endogenous immune effectors capable of targeting heterogeneous tumor populations [16]. Consequently, the rational design of modern immunotherapies has shifted from empirical engineering to computational optimization. This review presents a bioinformatics-guided design framework organized as a sequential, end-to-end workflow: (1) tumor profiling and antigen discovery using multi-omics integration and single-cell transcriptomics; (2) antigen prioritization and epitope validation using MHC-prediction tools applied as safety screens for conventional CARs and as neoepitope discovery platforms for TCR-like constructs; (3) CAR structural optimization through AlphaFold-based folding predictions and SnugDock docking simulations; (4) IL-18 engineering and delivery circuit design using NFAT-inducible, SynNotch, and post-translational Granzyme B-cleavage systems; (5) in silico safety prediction using Expitope 2.0 and STRING network analysis; and (6) preclinical validation in xenograft and syngeneic tumor models. Each of these stages is discussed in sequence across the following sections, with the goal of demonstrating how bioinformatics tools function not as isolated resources but as an integrated, iterative pipeline for the rational design of IL-18 armored CAR-T cells.

## Biological role of IL-18 in anti-tumor immunity

### IL-18 signaling pathways

The immunomodulatory effects of IL-18 begin at the cell surface, where it interacts with a conserved heterodimeric receptor complex comprising IL-18Rα and IL-18Rβ [17]. The formation of this high-affinity complex initiates an intracellular signaling cascade by recruiting the MyD88 adaptor protein [17]. This activates downstream kinases, including IL-1 receptor-associated kinases and tumor necrosis factor receptor-associated factor 6 (TRAF6), leading to the nuclear translocation and activation of transcription factors such as NF-κB, activator protein 1 (AP-1), and mitogen-activated protein kinases (MAPKs), including p38 and JNK [17]. Notably, synergy with IL-12 is clinically relevant, as IL-12 upregulates the IL-18 receptor and signals via STAT4, which operates in tandem with IL-18’s MyD88/NF-κB/AP-1 cascade to synergistically drive IFN-γ production [17, 18]. This coordinated signaling directs T cells toward a Th1 phenotype, which is essential for generating cytotoxic anti-tumor responses and producing memory T cells [18, 19].

### Effects on tumor microenvironment

In an immunosuppressive tumor microenvironment (TME), IL-18 is a key regulator that counteracts immune exclusion, transforming ‘cold’ tumors into ‘hot’ ones [20]. It induces chemokines like CXCL9, CXCL10, and CCL5, which promote T cell trafficking and infiltration into solid tumors [21]. Simultaneously, IL-18 reprograms myeloid populations, promoting the polarization of tumor-associated macrophages toward an M1 pro-inflammatory phenotype [22]. Additionally, IL-18 enhances NK cell cytotoxicity, stimulates dendritic cell maturation, thereby supporting antigen cross-presentation and facilitating epitope spreading, both of which are critical for anti-tumor immunity [23]. Together, these mechanisms counteract immunosuppression by reducing the numbers and activity of regulatory T cells and myeloid-derived suppressor cells (MDSCs), thereby restoring immunosurveillance and tumor regression (5,6).

### Safety considerations

Despite IL-18’s potent anti-tumor effects, its clinical use is limited by the risk of systemic toxicity [18, 25]. Uncontrolled systemic release of IL-18 can trigger severe hyperinflammatory reactions, including cytokine release syndrome (CRS) and macrophage activation syndrome (MAS) [25]. IL-18 binding protein (IL-18BP) serves as a physiological regulator by neutralizing mature IL-18 and limiting excessive inflammation [18, 26]. To safely use IL-18 in adoptive cell therapies, recent engineering strategies have focused on confining cytokine activity to TME [18, 25]. These include inducible promoter systems, such as NFAT-responsive elements, that tightly couple IL-18 transcription to CAR-mediated antigen engagement at the tumor site [18]. While transcriptional safeguards provide some spatial control, they risk baseline leakiness [25]. A better approach uses post-translational control by engineering pro-IL-18 so that its caspase-1 site is replaced, thereby activating it only by T-cell granzyme B [25]. This strictly restricts IL-18 maturation to the tumor site [27]. Combined, these strategies allow targeted anti-tumor immunity while minimizing the risk of systemic toxicity [27].

## Bioinformatics-guided CAR design

The bioinformatics workflow described in this section follows the sequential design logic introduced above: each stage builds directly on the outputs of the preceding one, beginning with tumor antigen identification and ending with in silico safety triage before any construct enters preclinical testing.

### Target antigen selection

The rational design of CAR-T cell therapies relies heavily on bioinformatics platforms to overcome tumor heterogeneity and prevent fatal off-target toxicities [4]. Target discovery requires the integrative interrogation of large-scale multi-omics databases, specifically. The Cancer Genome Atlas (TCGA) and the Genotype-Tissue Expression (GTEx) project to contrast malignant and healthy tissue transcriptomes systematically [28, 29]. When cross-referenced with the Human Protein Atlas, these datasets enable researchers to apply stringent differential expression thresholds, thereby computationally filtering out suboptimal candidates and isolating highly specific tumor surface antigens [30]. To resolve the immense complexity of intratumoral heterogeneity, single-cell RNA sequencing (scRNA-seq) maps diverse clonal subpopulations and their unique transcriptional activities. Concurrently, spatial transcriptomics delineates the physical architecture of the tumor microenvironment, identifying accessible target niches that facilitate optimal CAR-T cell infiltration and engagement [31, 32]. In the context of cold solid tumors, this multi-omics filtering is particularly critical for identifying conserved target antigens that justify the co-delivery of an IL-18 payload to remodel the surrounding immunosuppressive niche.

### Epitope prediction and validation

Precise epitope targeting is subsequently validated through robust in silico pipelines [33]. Putative antigenic regions are computationally predicted utilizing neural network-based algorithms like NetMHCpan and extensive databases such as the Immune Epitope Database (IEDB) [34], which evaluate peptide-MHC binding affinities and presentation propensities [35, 36] It is important to clarify the context in which MHC-prediction platforms such as NetMHCpan apply to CAR-T design. Conventional CAR-T cells recognize intact cell-surface antigens in an MHC-independent manner through their scFv domain, and do not rely on peptide-MHC presentation for target engagement. Therefore, NetMHC-based tools are not used to identify tumor antigens for standard CARs. Instead, their role in this design pipeline is two-fold: first, as an in silico safety screen to ensure that engineered scFv sequences, synthetic linkers, or junction regions do not inadvertently generate novel peptide sequences capable of binding host MHC molecules and eliciting unwanted immune responses; and second, as the primary antigen discovery tool in the specialized context of TCR-like CARs and peptide-MHC targeting constructs, which are designed explicitly to recognize MHC-presented neoantigens. To optimize target recognition, structural docking simulations such as RosettaDock [37] and SnugDock [38] map the free energy landscapes of single-chain variable fragment (scFv) antigen interactions, utilizing Monte Carlo sampling to predict binding orientation and affinity. Advanced homology modeling further predicts epitope immunogenicity and potential cross-reactivity [39]. Crucially, these conformational analyses account for steric constraints like steric shielding by glycans, as precise glycosylation sites fundamentally dictate epitope accessibility and the kinetics of antibody-antigen recognition [40–42].

### CAR construct optimization

The structural architecture of the CAR construct is iteratively refined using deep learning and physics-based models. Cutting-edge artificial intelligence, including AlphaFold and Rosetta algorithms, accurately predicts protein folding, thermodynamic stability, and complex 3D structures directly from primary sequences [43, 44]. Practically, this allows for a pipeline where AlphaFold3 resolves the 3D structures of the scFv and tumor antigen, followed by SnugDock simulations to calculate binding free energy (Delta G) and rationally introduce affinity-tuning point mutations. This atomic-level structural insight directs linker engineering, where the flexibility and length of hinge regions (e.g., utilizing standard (G4S)3 linkers) are explicitly tuned to optimize signaling geometry and enhance proper immune synapse formation. Furthermore, optimizing the intracellular signaling architecture including the precise stacking of costimulatory domains such as CD28 is vital to augment primary and secondary signal cascades, thereby maximizing CAR-T cell proliferation, survival, and anti-tumor cytokine production [45–47]. Beyond classical costimulatory stacking strategies, recent work has explored alternative intracellular signaling architectures to improve the therapeutic index of CAR-T cells. Notably, a recently published study demonstrated that replacing the conventional CD3zeta-based signaling module with a ZAP70-based CAR signaling architecture significantly attenuated cytokine release while preserving robust antitumor cytotoxicity [48]. This finding is particularly relevant in the context of IL-18 armored CARs, where managing the combined inflammatory output of both CAR activation and cytokine armoring is a core safety challenge. ZAP70-based signaling may therefore represent a complementary strategy to the inducible promoter and post-translational control systems, offering an additional layer of functional fine-tuning at the level of the signaling domain itself. Integration of such alternative architectures into the bioinformatics-guided design workflow for instance, through in silico modeling of ZAP70 signaling kinetics alongside NFAT activation thresholds warrants further investigation.

### In silico safety screening

Preclinical validation demands rigorous in silico safety screening to preemptively identify off-target effects. Computational tools predict off-tumor toxicities by evaluating target expression profiles across healthy tissues to establish critical safety margins [49]. Specifically, platforms like Expitope 2.0 cross-screen candidate scFv sequences against healthy proteomes, while STRING network analysis maps downstream signaling pathways to predict and mitigate systemic off-tumor toxicities. Network-based systems biology analyses mitigate unintended pathway activations, ensuring minimal disruption to healthy cellular networks [50]. Finally, comprehensive risk assessments evaluate HLA-binding promiscuity and potential cross-reactivity by analyzing structural similarities to known self-antigens. This stringent computational triage ensures engineered constructs achieve potent, tumor-restricted cytotoxicity with maximal clinical safety [51]. **(**Table 1**)**

**Table 1:** General Bioinformatics Tools Used in CAR-T and Immunotherapy Design

Category	Tool	Purpose	Output	Specific Application to IL-18 Armored CAR-T Design
Target Antigen & Epitope Prediction [52]	NetMHCpan / NetMHC	Utilizes artificial neural networks to predict the binding affinity of aberrant peptides to MHC class I and II molecules.	A ranked list of predicted neoantigens and binding affinity scores.	Identifies optimal, highly conserved tumor antigens while mapping the immunosuppressive tumor TME to justify the need for an IL-18 armor payload.
Structural Modeling [53]	AlphaFold (AlphaFold2, AlphaFold3)	Predicts the 3D structure of proteins and the joint structure of antigen-receptor complexes directly from primary amino acid sequences.	High-accuracy 3D structural models of proteins and biomolecular interactions.	Produces high-resolution tertiary structural simulations of the neoplastic antigen, the therapeutic scFv, and the armored IL-18 payload to authenticate conformational fidelity and spatial configuration.
Structural Modeling & Docking [54, 55]	Rosetta (RosettaAntibody, SnugDock	Performs high-precision 3D modeling, flexible docking simulations, and thermodynamic stability predictions for antigen-receptor interactions.	3D docking models, predicted changes in binding free energy, and stability optimization suggestions.	Optimizes the binding affinity between the CAR’s scFv and the tumor antigen; models the structural stability of the IL-18 payload to ensure it effectively binds to IL-18R.
In Silico Safety Screening [56]	Expitope 2.0	Assesses immunotherapeutic antigens for potential cross-reactivity and off-target toxicity against naturally expressed proteins in healthy human tissues.	Predictions of potential side effects, off-target toxicity, and cross-reactivity risk.	Screens the selected tumor antigen and the engineered CAR scFv framework to flag and eliminate potential off-target cross-reactivity with normal tissues.
Transcriptomic Profiling [57, 58]	Single-cell RNA sequencing (scRNA-seq) tools	Evaluates intratumoral heterogeneity, maps antigen distribution, and models CAR T-cell exhaustion mechanisms.	High-resolution mapping of diverse cellular subpopulations and identification of optimal target niches.	Maps the downstream signaling network of IL-18 release to ensure local immune activation (e.g., IFN-$\gamma$ production) without triggering systemic cytokine storms.
In Silico Safety Screening [59]	STRING / Network Analysis	Predicts unintended pathway activations by evaluating target expression profiles across healthy tissues to establish safety margins (from *In Silico Safety Screening* discussion).	Network-based pathway analysis to preemptively identify and mitigate off-tumor toxicities.	Evaluates the engineered CAR components, linkers, and IL-18 payload for immunogenicity, ensuring they won’t be recognized as foreign by the host immune system.

## Engineering IL-18 armored CAR-T cells

### Vector design

While lentiviral and gammaretroviral vectors are traditionally used to engineer T cells, their semi-random genomic integration poses significant risks of insertional mutagenesis and variable transgene expression [60]. To overcome these limitations, non-viral approaches are increasingly favored. The *Sleeping Beauty* transposon system utilizes a cost-effective “cut-and-paste” mechanism that offers a high genetic cargo capacity and a safer integration profile, and it has already been successfully deployed for the GMP-compliant manufacturing of IL-18-secreting CAR-T cells [61]. Furthermore, precision genome editing using CRISPR/Cas9 for site-specific integration is revolutionary [62, 63]. Directing the CAR knock-in to the TRAC (T-cell receptor alpha constant) locus simultaneously disrupts the endogenous TCR preventing graft-versus-host disease in allogeneic applications while placing the CAR under physiological promoter control to ensure uniform expression, avert tonic signaling, and delay immune exhaustion [64, 65].

### Controlled cytokine release strategies

Because constitutive secretion of pro-inflammatory cytokines like IL-18 can provoke severe systemic toxicity, tight regulatory circuits are essential. NFAT-responsive promoters couple IL-18 transcription directly to CAR activation, ensuring that cytokine release is strictly localized to the tumor microenvironment following target antigen engagement [66]. For enhanced spatial precision, SynNotch receptor systems employ dual-antigen “IF-THEN” logic gating: the recognition of a priming antigen by the SynNotch receptor cleaves and releases a transcription factor, which subsequently induces the expression of the CAR and/or therapeutic cytokines like IL-18 [67]. Additionally, small molecule inducible systems provide temporal control, functioning as tunable “ON/OFF switches” (via chemically induced dimerization or drug-stabilized degrons) to rapidly regulate T-cell activity or halt cytokine secretion in response to exogenously administered drugs [68]. Such tight dose control is clinically vital; unlike IL-12 or IL-15, which directly drive lethal systemic hyper-inflammation, IL-18 toxicity primarily manifests as macrophage activation syndrome (MAS) driven by localized IFN-gamma amplification loops [69]. Furthermore, engineered circuits must achieve sufficiently high local concentrations to outcompete circulating IL-18 Binding Protein (IL-18BP), a potent endogenous decoy receptor that binds mature IL-18 with high affinity and thereby attenuates systemic therapeutic activity [70].

### Co-expression systems

**Fig. 1:**
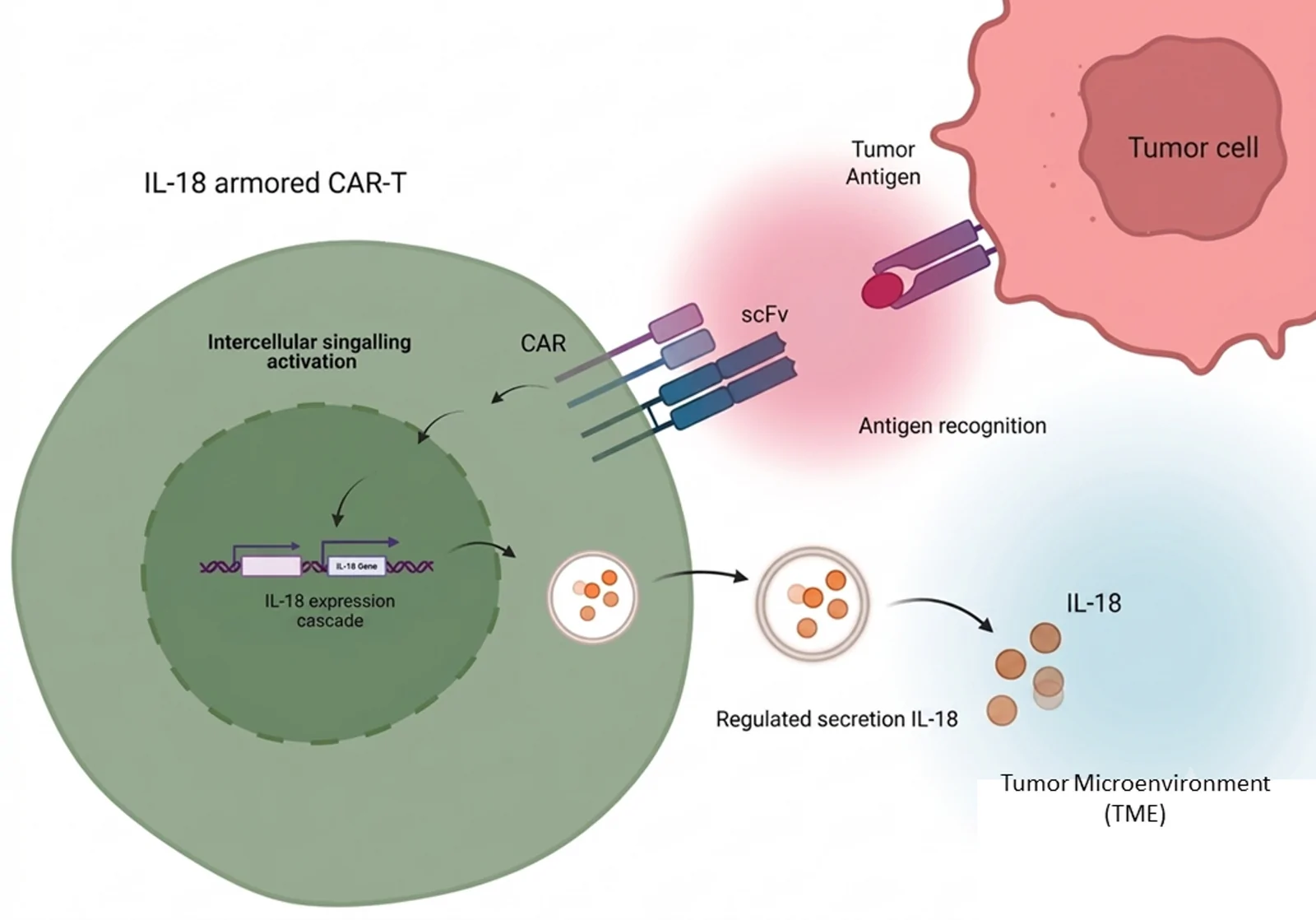
Structural design and expression system of IL-18 armored CAR-T cells

To ensure the simultaneous production of the CAR and the IL-18 payload from a single vector, polycistronic constructs typically utilize 2 A peptide-mediated ribosomal skipping (such as P2A or T2A) or IRES (internal ribosome entry site)-based translation [71]. These constructs incorporate specific secretory signal peptides to ensure the efficient cellular processing and export of active IL-18 directly into the tumor milieu [72–74]. However, moving these polycistronic systems toward clinical translation presents distinct manufacturing barriers, including genetic payload cargo size limitations within viral vectors and the chronic risk of accelerated T-cell exhaustion induced by localized IL-18 upregulation of immune checkpoints. A critical design decision within polycistronic IL-18 co-expression systems is whether IL-18 expression is constitutive or inducible. Constitutive expression, while simpler to engineer and more reliably quantifiable during manufacturing, carries meaningful safety risks: continuous IL-18 secretion in the absence of tumor antigen engagement can drive tonic immune activation, promote premature T cell exhaustion through chronic checkpoint upregulation, and generate systemic cytokine gradients that increase the risk of macrophage activation syndrome [75]. In contrast, inducible co-expression systems — in which IL-18 transcription is linked to CAR activation through NFAT-responsive promoters or SynNotch logic gates — restrict cytokine output to moments of genuine target engagement, substantially reducing off-tumor inflammatory risk. However, inducible designs impose additional vector payload demands, because the regulatory elements required for tight transcriptional control consume significant transgene capacity within standard lentiviral vectors, which typically accommodate inserts of approximately 8 kilobases. Larger payloads necessitate split-vector approaches or alternative delivery systems such as the Sleeping Beauty transposon, which has already demonstrated GMP-compliant feasibility for IL-18-secreting CAR-T manufacturing [75]. Taken together, the choice between constitutive and inducible IL-18 co-expression represents a fundamental design tradeoff between engineering simplicity, manufacturing scalability, and clinical safety that must be resolved on a construct-by-construct basis. Finally, the therapeutic potency of these armored cells can be further amplified through co-expression with checkpoint blockade modules. For instance, co-expressing PD-1 dominant-negative receptors (or chimeric switch receptors) shields the engineered T-cells from immunosuppressive signals, converting the hostile tumor microenvironment into a catalyst for sustained antitumor immunity [76, 77]. **(**Fig. 1**)**

## Mechanisms of cold tumor conversion

### Immune cell recruitment

The challenge in treating immunologically “cold” tumors is that effector lymphocytes cannot effectively penetrate the tumor bed [20]. IL-18 is a critical catalyst in overcoming this barrier by establishing localized chemotactic gradients that drive immune cell recruitment [21]. Engineered IL-18 secretion within TME strongly induces the production of key chemokines, most notably CXCL9, CXCL10, and CXCL11 [21, 22]. These serve as the primary ligands for the CXCR3 receptor, expressed on NK cells and activated effector T cells [21, 22]. By restoring the CXCL9/10–CXCR3 axis, IL-18 reverses chemokine dysregulation, guiding cytotoxic lymphocytes into tumor tissue [21, 22]. Successful immune infiltration also depends on T cells’ ability to breach the endothelial barrier, as solid tumors restrict lymphocyte entry [78, 79]. Olivera et al. demonstrated that IL-18, especially in synergy with IL-12, alters T cell surface O-glycosylation, creating core 2 O-glycans with sialyl Lewis X [78]. These E-selectin ligands enhance T cell adhesion, rolling, and extravasation across tumor endothelium [78, 79].

### Remodeling the tumor microenvironment

IL-18 remodels this environment by reprogramming immunosuppressive TAMs toward a pro-inflammatory M1-like phenotype with increased MHC-II and CD86 expression [20]. IL-18 shifts TAMs toward an M1-like phenotype with increased MHC-II and CD86 expression [20, 80]. This IL-18-triggered Th1 inflammation directly matures DCs, creating an immunopermissive environment vital for restoring antigen presentation machinery [17, 81]. IL-18 enhances the recruitment and maturation of DCs within the tumor, improving their capacity for antigen processing and presentation [17]. IL-18-induced local production of interferon-gamma (IFN-γ) upregulates major histocompatibility complex class I (MHC-I) on tumor cells and MHC-II on antigen-presenting cells. This comprehensive remodeling disrupts stromal tolerance and establishes an immunopermissive niche necessary for sustained clinical efficacy [20].

### Synergy with endogenous Immunity

**Fig. 2:**
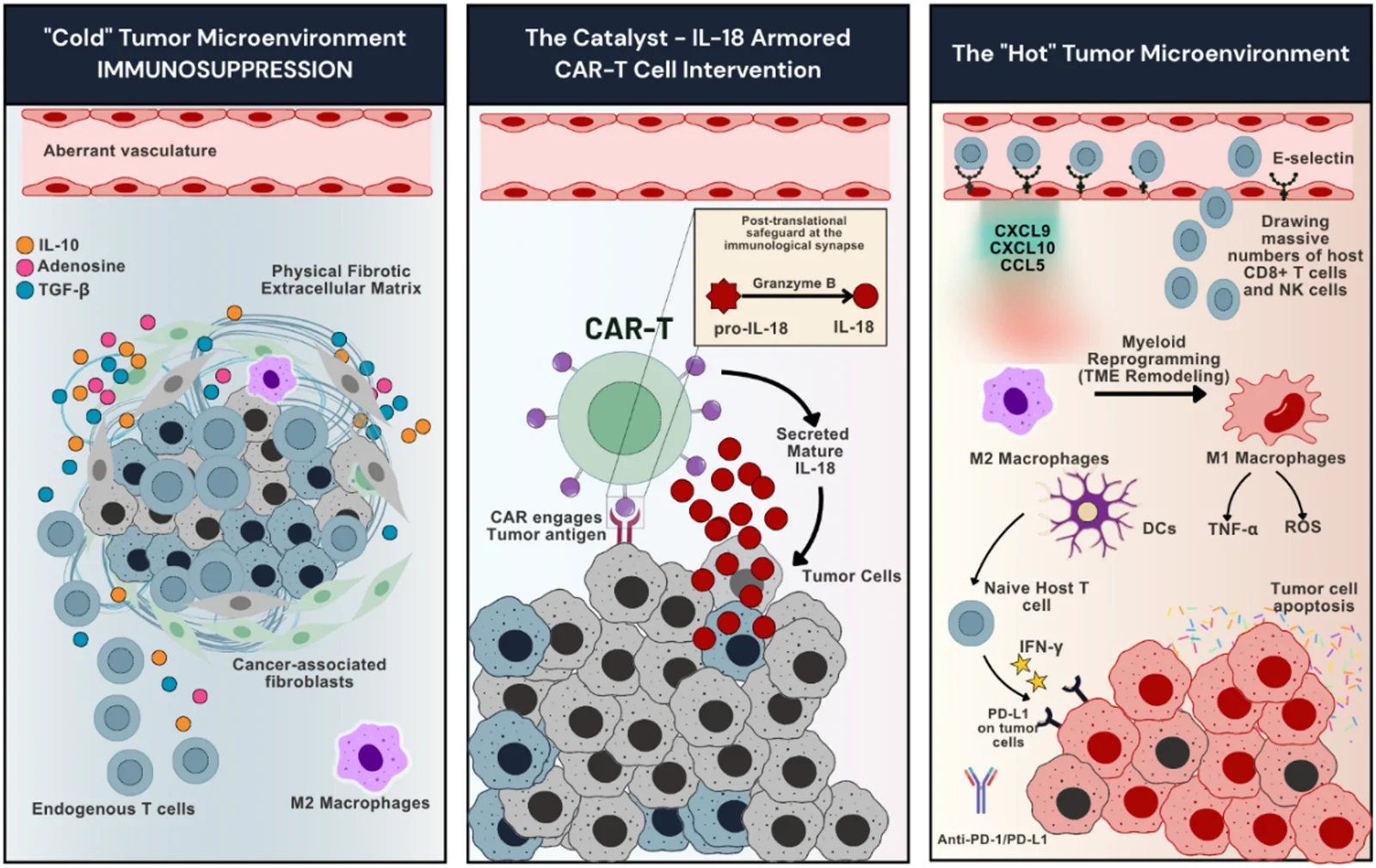
Mechanism of IL-18-armed CAR-T cells in converting cold tumors into hot tumors. ***(Left)*** *The “cold” tumor microenvironment actively suppresses and physically blocks immune cell entry. (Center) Upon tumor antigen recognition*,* engineered CAR-T cells safely release active IL-18 via Granzyme B cleavage directly at the immunological synapse.* ***(Right)*** *Local IL-18 converts the tumor to a “hot” state by recruiting host T and NK cells*,* reprogramming suppressive M2 macrophages into inflammatory M1 cells*,* and driving dendritic cell-mediated epitope spreading. The resulting IFN- release upregulates tumor PD-L1*,* making the tumor highly sensitive to checkpoint blockade therapies.* **CAR-T**, chimeric antigen receptor T cell; **CAF**, cancer-associated fibroblast; **CCL5**, C-C motif chemokine ligand 5; **CXCL9/10**, C-X-C motif chemokine ligand 9/10; **DC**, dendritic cell; **IFN-**, interferon-gamma; **IL**, interleukin; **M1/M2**, macrophage polarization states 1 and 2; **NK**, natural killer; **PD-1**, programmed cell death protein 1; **PD-L1**, programmed death-ligand 1; **ROS**, reactive oxygen species; **TGF-**, transforming growth factor-beta; **TME**, tumor microenvironment; **TNF-**, tumor necrosis factor-alpha

The therapeutic efficacy of IL-18-armored cellular therapies extends beyond the direct cytotoxic effects of the engineered cells, as IL-18 acts as a central mediator that activates a broad endogenous immune response [18, 80, 82]. One key mechanism is epitope spreading [80, 82]. IL-18-secreting T cells mediate tumor lysis, releasing neoantigens into the TME [82]. Cross-presented by dendritic cells, these antigens activate a broad CD8 + T-cell response for long-term immunity and relapse reduction [80]. IL-18 also boosts innate immunity by enhancing NK cell proliferation, cytolytic ability, and Fas ligand expression, providing secondary defense against tumor clones that evade T cell recognition by downregulating MHC-I. IL-18-driven IFN-γ production causes adaptive resistance by increasing immune checkpoint expression on tumor and myeloid cells [24]. While this temporarily protects the tumor, it creates a vulnerability that PD-1/PD-L1 blockade can exploit [18, 80]. Advanced bioengineering employs spatial control mechanisms, such as post-translational safeguards like Granzyme B-cleavable pro-IL-18 and NFAT-inducible promoters, to restrict IL-18 activation and release to the tumor bed [18, 83]. These measures ensure that synergy with the endogenous immune system achieves effective tumor eradication while preventing systemic hyperinflammation [83]. **(**Fig. 2**)**

## Preclinical evidence and translational safety

### In-vitro functional and cytotoxicity assessment

The in vitro functional analysis of CAR-T cells usually starts with cytotoxicity assays. The aim of such assays is to determine whether or not engineered T cells can kill target tumour cells. An actively dynamic measurement of cytolytic activity is provided by real-time impedance-based assays, including those measured using xCELLigence platform. This is achieved through observing change of electrical impedance as lysis of tumour cells occurs [84]. state that these assays possess several benefits over standard endpoint assays due to the fact that the assays can be used to monitor cytotoxicity continuously, in the absence of label usage. They have confirmed that the IL-18 armored CAR-T cells, specifically, can stimulate IFN and other cytokines, which suggests that they have elevated their functional activity [85].

### In vivo efficacy and tumor control models

Xenograft and syngeneic tumour models are the two models that are commonly used to determine the efficacy of the IL-18 armored CAR-T cells in vivo via clinical trials. By xenograft model, human CAR-T cells can be tested with respect to direct anti-tumor activity, an application that necessitates the presence of immune deficient mice (like NSG mice) although these are engrafted with human tumours. Syngeneic models are prepared by placing CAR-T cells of mouse cells (immune-competent mice) into the mice. The models provide a more insight into the interaction of these cells with the native immune system [85, 86]. **(**Table 2**)**

**Table 2:** Summary of preclinical studies evaluating IL-18 armored CAR-T cells

Model System	Antigen Target	Tumor Type	CAR Design	IL-18 Strategy	CAR Construct Features	Key Findings	Limitations
Xenograft and syngeneic mouse tumor models [85]	CD-19	Hematologic and solid tumor	2nd generation CAR-T	Constitutive IL-18 secretion	Second-generation CAR-T cells engineered to secrete IL-18	Improved T-cell expansion, persistence and tumor killing; also stimulated endogenous immunity	Potential for systemic inflammation; poor translation to humans (mouse immune system is less complex than humans)
Syngeneic and xenograft mouse models [87]	DLL3	Small cell lung cancer	IL-18 secreting CAR-T cells	Constitutive IL-18 secretion	Engineered CAR-T cells to secrete IL-18	Enhanced T-cell proliferation, IFN-γ secretion, and anti-tumor efficacy	Risk of cytokine-related toxicity, immune-mediated side effects
Murine solid tumor models [88]	Macrophage associated targets	Solid tumor	CAR-T platform	Inducible IL-18 expression	CAR-T cells expressing inducible IL-18 (TRUCK design)	Increased T-bet⁺ effector cell differentiation and enhanced killing of established solid tumors	Poor clinical translation; difficult to control cytokine secretion
Preclinical solid tumor models [89]	EGFR	Triple-negative breast cancer and other solid tumors	4th generation armored CAR-T	NFAT- driven inducible IL-18	CAR-T cells with antigen-inducible IL-18 system	Tumor-specific activation, higher anti-tumor activity and lower toxicity	Complex engineering; preclinical
Tumor-dependent preclinical models [90]	CAR αβ	Solid Tumor	2nd generation armored CAR-T	Granzyme B-activated IL-18	CAR-T cells releasing IL-18 when activated by enzyme B	Tumor-specific release of cytokines to enhance safety and efficacy	Preliminary results; needs further validation

### Safety profiling and translational control strategies

Safety profiling is necessary in order to achieve or significantly advance translational development of armored CAR-T therapies. Cytokine release syndrome (CRS), which is typified by an inflammatory outcome, which is mediated by IL-6 persists as the most notable presentation of toxicity which is linked to CAR-T treatment [91]. state that management can often imply the usage of tocilizumab to prevent IL-6.

The other significant side effect is called Immune Effector Cell-Associated Neurotoxicity Syndrome (ICANS). Recently, it has been implied that, in addition to interleukin-6 (IL-6), interleukin-18 (IL-18) may also be possible mediators or biomarkers of the risk of neurotoxicity [92].

On-target/off-tumor toxicity occurs when the target antigen is expressed in normal tissues with relatively low levels, and is targeted by CAR-T cells. This effect is regularly tested on preclinical models through humanized xenografts and syngeneic systems [84]. Lastly, once IL-18 armored CAR-T cells get closer to clinical applications, the safety and consistency of the products needs to be safeguarded by following the Good Manufacturing Practice (GMP) in the production and performing the pre-clinical trials thoroughly. These involve the observation of the biomarker profiles and dose escalation [93].

## Integration with multi-omics approaches

### Single-cell RNA sequencing

Single-cell RNA sequencing (scRNA-seq) resolves heterogeneity of engineered CAR-T cells [94, 95]. This allows precise mapping of CAR-T cell states, distinguishing among highly active effector subsets, memory T cells, and terminally exhausted cells [95]. Clinical endpoints such as sustained remission correlate with TCF7/LEF1-expressing memory T cells. scRNA-seq detects T-cell dysfunction signatures upregulation of LAG3, TIM3, TOX, and NR4A that predict premature CAR-T failure [95]. Moreover, scRNA-seq is essential for profiling the tumor ecosystem [95]. It deconvolutes cellular interactions within the TME and accurately maps immunosuppressive networks, including Tregs and M2 macrophages [95, 96]. This high-dimensional mapping is important for developing targeted therapies to convert immunologically “cold” tumor ecosystems into inflamed environments [95, 96].

### Proteomics and secretomics

While transcriptomics defines cellular potential, proteomics and secretomics provide the direct roles of effector molecules governing CAR-T cell cytotoxicity and systemic safety [97]. Advanced platforms such as mass cytometry (CyTOF) and mass spectrometry allow detailed analysis of cytokine and chemokine networks to predict severe toxicities [95]. Longitudinal serum proteomics can signal cytokine release syndrome (CRS) and neurotoxicity syndrome (ICANS) by detecting early elevations of IL-6, IFN-γ, and angiopoietin-2 (ANG2) [98]. Moreover, global phosphoproteomics is crucial for understanding CAR regulation [97]. Analyses reveal that divergent costimulatory domains, such as CD28 and 4-1BB, show distinct phosphorylation patterns in signaling pathways (e.g., LCK/ZAP70 activation) [97]. Understanding these pathways allows optimization of CAR activation for effective tumor destruction [97].

### AI-driven optimization

Translating massive multi-omics datasets into precision oncology requires artificial intelligence [99]. Supervised machine learning algorithms, such as random forests and extreme gradient boosting (XGBoost), are increasingly used to predict CAR efficacy and toxicity [95]. For example, ensemble models combine biomedical knowledge graphs with cytokine profiles to accurately predict CRS onset and severity, enabling preemptive management of adverse events [98, 100]. AI-driven digital twin models of tumor–immune interactions use a patient’s multi-omics and clinical data to simulate CAR-T cells, malignant cells, and the TME [99]. These models serve as virtual trials, allowing patient-specific interactions to optimize personalized treatment plans [99]. Moreover, generative AI and deep learning platforms such as AlphaFold are transforming CAR design by rapidly predicting protein structures and signaling motifs, thereby accelerating the rational engineering of high-affinity CAR constructs [82, 101, 102]. **(**Table 3**)**

**Table 3:** Comparison of cytokine-armored CAR-T cell strategies

Feature	IL-18 CAR-T	IL-12 CAR-T	IL-15 CAR-T
Primary immune function [19, 23, 83]	Broad TME immune remodeling	Potent Th1 effector polarization	Enhanced survival and persistence
Major signaling pathway [23, 103]	MyD88, NF-κB, AP-1	JAK and STAT4 cascades	STAT5 and PI3K-AKT
Induced T-cell phenotype [23, 25, 80, 82]	Effector memory, less exhausted	Potent terminal effector cells	Stem-like central memory
Systemic toxicity risk [25, 82, 104]	Moderate CRS/MAS risk	High, often fatal toxicity	Manageable CRS, excessive proliferation
Bystander cell activation [23, 80, 83]	Recruits NKs, M1 macrophages	Reactivates macrophages and NKs	Expands endogenous NK cells
In vivo persistence [23]	High remodels the TME	Low risks of terminal exhaustion	Very high, preserves stemness

**Fig. 3:**
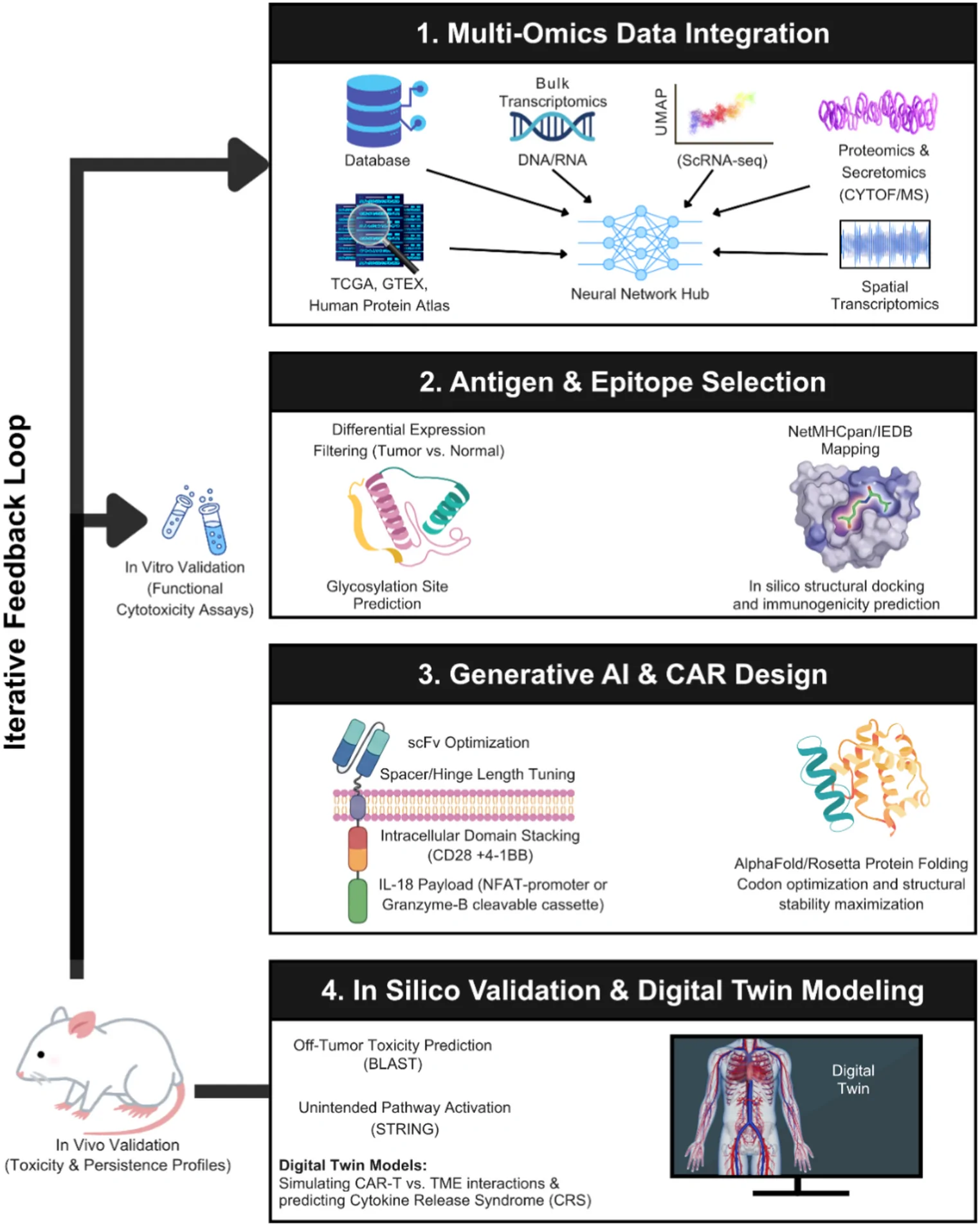
Bioinformatics workflow for designing IL-18 armored CAR-T cells. ***(1)*** *Multi-omics data integration comprehensively profiles the tumor ecosystem.* ***(2)*** *Computational algorithms select highly specific surface antigens and perform structural epitope mapping to identify accessible extracellular binding domains for the CAR scFv* ***(3)*** *Generative AI optimizes the 3D structure of the CAR construct and the IL-18 payload.* ***(4)*** *In silico digital twins simulate tumor-immune interactions to predict safety and toxicity. An iterative feedback loop continuously refines the predictive AI models using empirical in vitro and in vivo validation data*

These integrated multi-omics and AI-driven approaches collectively enable a rational, data-guided framework for optimizing CAR-T cell design and function **(**Fig. 3**)**. The workflow highlights how bioinformatics pipelines translate complex tumor-immune data into precise engineering of IL-18 armored CAR-T cells.

## Challenges and future directions

Limited penetration of engineered T cells into tumour areas is one of the most important challenges CAR-T cell therapy must deal with when it comes to the treatment of solid tumours. To a great extent, this phenomenon can be attributed to the thick stroma and unhealthy vasculature which are typical of a significant number of solid tumours. These properties form a physical obstacle impeding the trafficking and extravasation of immune cells in their presence [105, 106].

Additionally, it should be mentioned that there is a considerable immunosuppressive effect observed by the tumour microenvironment (TME). This is mostly due to the occurrence of different factors, such as transforming growth factor β (TGF- 10), interleukin (IL-10) and adenosine. Due to the viability of activation of CAR-T cells, proliferation, and cytotoxic activity induced by these molecules, its curative results are not optimal as they could be. To reverse the effects of the TME-based suppressive action, some measures are under study. The engineering of CAR-T cells to evade TGF-β signaling has been demonstrated as one of these approaches by [107, 108].

The technical challenge is substantial as the production of controlled and accurate expression IL-18 into armored CAR-T cells is a major task. Excessive release or dysregulated release of IL-18 can lead to systemic inflammation and toxicity, but low expression may not be capable to enhance the efficacy of the therapeutic interventions. Even though research and development is going on in regards to the creation of inducible or tumor-restricted IL-18 expression systems, there remains a problem of achieving consistent safety profiles [109, 110].

One of the possible future directions in CAR-T therapy of solid tumours is the combination of CAR-T cells with other modalities, including immune checkpoint blockade (like anti-PD-1/PD-L1 antibodies) and oncolytic viruses. These combination strategies are aimed at enhancing anti-tumor responses by circumventing immunosuppressive responses and encouraging the lysis of tumour [111].

Lastly, the creation of novel CAR systems (including logic-gated CARs (activation in response to multiple signals) and universal allogeneic platforms (off the shelf capabilities) has a tremendous potential to increase accessibility and safety. They note that these innovations are aimed at enhancing the access to patients through the standardized cell products, which are readily accessible. Also, they seek to minimize the chances of side effects and off-tumor toxicity through on-target delivery [105, 106].

## Conclusion

IL-18 armored CAR-T cells represent a biologically grounded and computationally guided approach to overcoming the barriers that render solid tumors resistant to immunotherapy. By combining cytokine-mediated TME remodeling with precise antigen targeting, controlled release engineering, and multi-omics-informed design, this strategy addresses both the immunological and structural dimensions of cold tumor exclusion. Emerging AI-driven tools further accelerate construct optimization and safety prediction. While challenges related to consistent IL-18 expression, stromal penetration, and clinical translation remain, continued integration of bioinformatics with advanced cell engineering positions IL-18 armored CAR-T cells as a compelling candidate for next-generation solid tumor immunotherapy.

## Data Availability

No datasets were generated or analysed during the current study.

## Declarations

### Use of generative AI and AI-assisted technologies

No AI tools or technologies were used in the preparation of any part of this manuscript.
